# Perspective on Improving Environmental Monitoring of Biothreats

**DOI:** 10.3389/fbioe.2018.00147

**Published:** 2018-10-23

**Authors:** John Dunbar, Segaran Pillai, David Wunschel, Michael Dickens, Stephen A. Morse, David Franz, Andrew Bartko, Jean Challacombe, Timothy Persons, Molly A. Hughes, Steve R. Blanke, Robin Holland, Janine Hutchison, Eric D. Merkley, Katrina Campbell, Catherine S. Branda, Shashi Sharma, Luther Lindler, Kevin Anderson, David Hodge

**Affiliations:** ^1^Los Alamos National Laboratory, Los Alamos, NM, United States; ^2^Food and Drug Administration, Washington, DC, United States; ^3^Pacific Northwest National Laboratory, Richland, WA, United States; ^4^Battelle Memorial Institute, Columbus, OH, United States; ^5^Centers for Disease Control and Prevention, Atlanta, GA, United States; ^6^IHRC, Inc., Atlanta, GA, United States; ^7^Retired, Gettysburg, PA, United States; ^8^Government Accountability Office, Washington, DC, United States; ^9^University of Virginia, Charlottesville, VA, United States; ^10^University of Illinois, Urbana, IL, United States; ^11^Queen's University Belfast, Belfast, United Kingdom; ^12^Sandia National Laboratory, Livermore, CA, United States; ^13^Department of Homeland Security, Washington, DC, United States

**Keywords:** biowatch, aerosols (bio-), biological weapon attack, detection, real-time sensing

## Abstract

For more than a decade, the United States has performed environmental monitoring by collecting and analyzing air samples for a handful of biological threat agents (BTAs) in order to detect a possible biological attack. This effort has faced numerous technical challenges including timeliness, sampling efficiency, sensitivity, specificity, and robustness. The cost of city-wide environmental monitoring using conventional technology has also been a challenge. A large group of scientists with expertise in bioterrorism defense met to assess the objectives and current efficacy of environmental monitoring and to identify operational and technological changes that could enhance its efficacy and cost-effectiveness, thus enhancing its value. The highest priority operational change that was identified was to abandon the current concept of city-wide environmental monitoring because the operational costs were too high and its value was compromised by low detection sensitivity and other environmental factors. Instead, it was suggested that the focus should primarily be on indoor monitoring and secondarily on special-event monitoring because objectives are tractable and these operational settings are aligned with likelihood and risk assessments. The highest priority technological change identified was the development of a reagent-less, real-time sensor that can identify a potential airborne release and trigger secondary tests of greater sensitivity and specificity for occasional samples of interest. This technological change could be transformative with the potential to greatly reduce operational costs and thereby create the opportunity to expand the scope and effectiveness of environmental monitoring.

## Introduction

The release of a BTA as an aerosol has the potential to kill thousands of individuals (Riedel, 2004; Nicogossian et al., 2011). The initial phase of an undetected attack would be silent and unremarkable. Within a few days, however, the surge of victims and panic could overwhelm an unprepared public health system (Isukapalli et al., 2008), cripple key infrastructure, and create economic shockwaves. To avert such disasters in the United States, a stronger biodefense capability was developed under the guidance of Homeland Security Presidential Directives (HSPDs) and Congressional Acts. For example, the 2004 HSPD-10 identified four pillars of biodefense: (i) threat awareness; (ii) prevention and protection; (iii) surveillance and detection; and (iv) response and recovery (HSPD, 2008). The pillars emphasized the need for better global intelligence and interdiction of efforts to acquire bioweapons, a robust surveillance system to rapidly detect an attack, and rapid deployment of the strategic national stockpile and distribution and dispensation of medical countermeasures to reduce fatalities. The 2004 Bioshield Act (Jones et al., 2005; Russell, 2007) provided $5.6 billion to develop, procure, and stockpile Medical Countermeasures (MCMs) against high-consequence biological agents. Efficient distribution of the MCMs after an attack could reduce illness and fatalities, but such execution requires a surveillance system that can effectively detect, identify, and characterize the BTA and scale of an attack.

Surveillance for a biological attack currently relies on a combination of public health/clinical surveillance and environmental monitoring. Public health/clinical surveillance involves a combination of syndromic disease surveillance (Gould et al., 2017; Lall et al., 2017; Mathes et al., 2017; Ridgway et al., 2017) and conventional reporting of disease to local or state health departments. The development of FDA-approved, point-of-care multiplex diagnostics may facilitate accurate diagnoses and the timely reporting needed to release MCMs from the National Strategic Stockpile for post-exposure prophylaxis (Doggett et al., 2016). However, sole reliance on public health/clinical surveillance averts only a fraction of the health consequences because of the time delay in recognizing an attack (Kaufmann et al., 1997; Wein et al., 2003). Even if the first symptomatic cases resulting from a covert attack are diagnosed expeditiously, the scale and scope of the exposure event would be unknown. The scale and impacted area must be determined to guide the distribution and dispensation of MCMs. When the number of identified cases surges enough to indicate a large-scale exposure event, crucial time for consequence management has been lost. This limitation motivates the use of complementary environmental monitoring. Environmental monitoring has the potential to detect an attack prior to disease onset, enabling accelerated release of appropriate MCMs for post-exposure prophylaxis and disease mitigation.

The Biowatch program was implemented in 2003 for the purpose of detecting large-scale biological attacks (Shea and Lister, 2003; National Academy of Sciences, 2013). It represents the current state-of-the-art for environmental monitoring of BTAs. The current program monitors outdoor air at selected sites for the presence of a subset of BTAs in more than 30 cities across the U. S. Monitoring involves the collection of airborne particles on membrane filters over a 24-h period, although shorter collection periods have been used on occasion. After collection, the filters are removed and transported to a Biowatch laboratory for analysis, which consists of the extraction of nucleic acids followed by semi-quantitative real-time PCR amplification and detection of target sequences associated with the BTAs of interest. Based on the current sample processing and testing scheme, results are generally available between 12 and 36 h after airborne particles are collected on an air filter. During the past 12 years of the program's operation, numerous technical and operational challenges of city-wide, outdoor bioaerosol monitoring have become apparent. For example, detection of a BTA can be problematic due to dilution effects as a plume of aerosolized material travels and mixes with surrounding air (Craft et al., 2005; Stuart and Wilkening, 2005; Buckeridge et al., 2006; Nicogossian et al., 2011). The ability of a given particle collector to collect an aerosolized agent can also vary widely because city-wide airflow patterns are highly variable. Changes in the direction and velocity of wind throughout the day, changing weather conditions, fluctuation in microclimate conditions in complex urban landscapes, changes in human activity (e.g., traffic) patterns, and associated variability in the background chemical and biological composition of ambient aerosols (Jones and Harrison, 2004; Fahlgren et al., 2010; Franzetti et al., 2011; Bertolini et al., 2013; Bowers et al., 2013) can affect the capture and detection of agents of concern. These phenomena can create variation in the probability of sufficient target capture by individual collectors, underscoring the need for a dense network of outdoor collectors to reliably detect a biological attack. Related operational challenges include the cost of labor to retrieve and transport the captured samples (daily or more often) from the network of aerosol collectors, transport the samples to a Biowatch laboratory, and process them for detection of BTAs. The total operational cost is estimated at >$400/day per detector (i.e., $87 M operation budget for 600 collectors over 365 days) (Person and Currie, 2015). The cost vs. benefit of the program has been increasingly challenged, which motivates a re-examination of current approaches for environmental monitoring.

With this foundation, a panel of 39 subject matter experts from federal agencies, academia, and industry (participant list included at the end of this article) met to discuss the current state-of-the-art technology for 9 h over three consecutive days during a conference on chemical and biological terrorism defense. The participants included past and present DHS program managers tasked with Research and Development to support the Biowatch program, experts who have contributed in various ways to environmental monitoring of BTAs over the entire history of the Biowatch program, and members of other U.S. Government agencies that have participated in prior assessments of the Biowatch program. State government end-users of the Biowatch program did not attend the scientific conference from which this discussion group was assembled. The discussion group was tasked with providing recommendations to address three specific challenges: value and benefit to public health, cost and sustainability, and probability of detection. The consensus recommendations from the discussion group include operational and technological changes that range from incremental to transformative. Implementing these recommendations could lead to a more robust and cost-effective environmental monitoring program.

A crucial starting point was the recognition of two operational modes for monitoring systems intended to provide early warning of a biological attack. The two modes are conventionally referred to as *detect-to-warn* and *detect-to-treat*. The two operational modes were originally defined in the context of warning systems for U.S. military personnel (National Research Council, 2005). However, in a civilian environment, a much higher standard of evidence is required to initiate public health actions. For example, detect-to-warn in the military context is intended to allow military personnel to avoid an approaching bioaerosol cloud or to enable distribution of prophylactic antibiotics to military personnel when a possible exposure is suspected, but such actions are unrealistic for civilian populations. Continued use of the military definitions of biosurveillance operational modes sets a performance expectation for civilian warning systems that is difficult or impossible to achieve with the current technology. Therefore, we redefined the operational modes for compatibility with a civilian context. Our perspective of key distinctions between the military and civilian contexts is outlined in Table 1. Under the definitions for civilian use that we employ here (detailed below), the two operational modes for environmental biomonitoring have different evidence requirements for implementation of public health actions (Table 2).

**Table 1:** Detection contexts and modes.

	**U.S. military**^**a**^		**Civilian**	
	**Detect-to-warn**	**Detect-to-treat**	**Detect-to-warn**	**Detect-to-treat**
Objective	Minimize exposure/Expedite treatment	Expedite treatment	Expedite diagnosis	Expedite treatment
Time-of-action-after-release	Minutes	Minutes-to-hours	1–2 days	Multiple days
False alarm acceptability	Yes	Yes	Yes	No
Population	Homogenous (healthy young adults)		Heterogenous (all health states and ages)	

a *Derived from National Research Council (2005)*.

**Table 2:** Evidence standards for early detection modes.

**Evidence**	**Detect to warn**	**Detect to treat**
**AGENT DETECTION**		
Initial positive	+	+
Confirmatory positive - same sample, additional orthologous assays	+	+
Positives from other location(s)	–	+
**AGENT CHARACTERIZATION**		
Not-near neighbor	+	+
Not natural occurrence	+	+
Viable	–	+
Drug susceptibility	–	Desired
**EVENT CHARACTERIZATION**		
Approximate exposure area	–	+
**OPERATIONAL CONCLUSION**		
“Agent detected”	+	N/A
“Attack highly likely, viable agent, exposure zone estimated”	N/A	+

The detect-to-warn mode simply alerts public health officials of a *possible* large-scale exposure event so that identification of early cases of illness may be faster and more reliable (i.e., the potential for misdiagnosis may be reduced if physicians are alert for specific BTAs). In the detect-to-warn mode, the early cases of illness diagnosed through conventional public health channels provide confirmation of an exposure event. The early cases can also provide additional characterization of the event and the identification of the potential exposure zone that can be used to trigger a larger public health response for soon-to-emerge cases. Detect-to-warn is the easiest mode to implement. Current environmental monitoring for airborne BTAs operates in the detect-to-warn mode (using the definition provided here) but lacks some of the critical elements needed to support a rapid response, namely adequate data or demonstrated approaches to gather supplemental data that can engender widespread confidence among public health officials.

The detect-to-treat mode alerts public health providers of a *high-confidence* exposure event and can trigger population-level distribution of MCMs for post exposure prophylaxis if the scale and geographic scope of the attack is reasonably understood. In this mode, the presence of an air borne biological agent of interest must be established with high confidence because MCMs (i.e., antimicrobials and vaccines) cannot be delivered to a population based on inconclusive or false positive results. Population-level treatment for an unconfirmed threat is too costly in logistics, materials, and possible adverse health events. Adverse health events (including fatalities) can occur as a result of potential side-effects or adverse interactions with other medications in a large, physiologically heterogeneous civilian population in which an unknown number may also have varying degrees of immune-suppression.

A major problem for either operational mode is the possibility of detecting naturally occurring environmental organisms (e.g., *Bacillus anthracis* or *Francisella tularensis*) or genetic near neighbors that are indistinguishable from the agent of interest by the assays employed. For example, the microorganisms that cause anthrax and tularemia are endemic in the U. S. and can be found in environmental matrices such as soil and water, respectively, that can serve as sources of low numbers of airborne organisms. However, the airborne concentrations or the viability of these naturally occurring agents are likely insufficient to cause human disease as suggested by the absence of reported cases of inhalation anthrax and pneumonic tularemia in areas where Biowatch is deployed. This is particularly significant with *F. tularensis* because the infectious dose may be <10 organisms (Jones et al., 2005). Close relatives (or genetic near neighbors) of these microorganisms (particularly *F. tularensis*) may occasionally be found on air collection filters and detected by current assays resulting in false positive results. The Biowatch program reported 149 false positives from 2003 to 2012 (Person and Currie, 2015). The occasional presence of a close relative of a biological agent of interest in air is particularly problematic for detect-to-treat environmental monitoring because it increases the data required to distinguish a natural phenomenon from a biological attack, or it forces the use of thresholds that define a “positive” as a sample containing an exceptionally large quantity of target material that is never seen in natural aerosols. Additional data to define a “positive” may include coordinated detection by widely dispersed monitors, more extensive and accurate genetic analysis of the captured organism, and demonstration of viability of the organism. These technical hurdles were recurring points of discussion by the panel of experts in the course of developing recommendations for improved or next generation environmental monitoring systems.

### Recommendation 1–boost stakeholder confidence by a thorough validation of system efficacy

Lack of confidence by stakeholders (e.g., public health officials) in the performance of environmental monitoring is a significant challenge. Like any clinical diagnostic device, the end-to-end performance of an environmental monitoring system must be thoroughly validated under relevant conditions and the benefit must be unambiguous (Person and Currie, 2015). Each step of the monitoring process has factors that can affect the overall performance of the system. Inadequate validation, uncertainty about false positive and false negative rates, lack of clarity about the probability of detection (as a function of agent quantity and the numerous factors that affect system performance), insufficient event characterization, and uncertainty about degree of consequence management mitigation engender skepticism of the value of environmental biomonitoring. Consequently, an end-to-end, statistically rigorous performance assessment of currently fielded collection devices and their associated sample processing and analytical methods is needed. Developing performance standards in partnership with stakeholders should be considered. End-to-end assessments offer the opportunity to understand and explore possible improvements associated with collection, stability, recovery, and analysis of target analytes. Rigorous study design, sample selection, and statistical analysis can provide confident and relevant estimates of false negative and false positive rates that provide decision makers with crucial information.

### Recommendation 2–develop a different approach to sample collection to simplify sample processing

The use of conventional filters for the collection of aerosol samples poses several problems for the efficient detection of biological agents. First, the pore size of conventional filters is ill-suited to capture cell-free, non-aggregated protein toxins. This leaves a gap in surveillance of BTAs of interest. Second, conventional filters are not compatible with automated procedures for the extraction and detection of molecular signatures from biological agents. The filters used in the Biowatch program, for example, must be manually cut and folded to fit into tubes for sample processing. The requirement for manual filter-processing increases logistical and cost burdens while also creating potential for cross contamination of samples. Potential alternatives for conventional filters include dissolvable filters and liquid aerosol collection. However, these alternatives need to be further evaluated to determine if they support the intended use and application.

Dissolvable filters offer a near-term solution to the processing obstacles encountered with the conventional filters that are currently used for aerosol sample collection. Water-soluble filter materials (e.g., gelatin; Burton et al., 2007 or alginate; Lauer and Masters, 1988) are available but are problematic because (a) they can dissolve during aerosol collection under conditions of high humidity and, (b) they can render sample processing buffers too viscous. A recently developed filter material that dissolves in high concentrations of a chaotropic salt solution is currently being evaluated for use in forensics applications and pathogen detection systems (Luna Inc)^1^ and should be evaluated as a replacement for conventional filters used for aerosol collection.

Liquid collection is another potential solution for autonomous detectors designed to perform both aerosol collection and sample processing. Liquid collection systems are problematic for long collection periods due to evaporation issues. However, if aerosol collection for environmental surveillance shifts to the short collection intervals needed for timely warning, liquid collection might be a compelling solution to simplify processing, improve toxin collection, and facilitate recovery of viable organisms if proper osmolarity conditions can be maintained.

Another area that may bring improved signature acquisition is the development of new filter materials or concentration approaches that improve the recovery of nucleic acid signatures. Loss of nucleic acid signatures during aerosol collection or sample processing is an ongoing concern for detection sensitivity. Loss of nucleic acid signatures may arise from degradation of the molecules during sample collection or from mechanisms that reduce signature recovery such as spontaneous cross-linking of glycosylated proteins to DNA or presence of co-collected materials that reduce the efficiency of DNA recovery during the extraction process. Approaches that stabilize RNA and DNA signatures and maximize recovery may increase the range of recoverable agents (e.g., RNA viruses) and their likelihood of detection.

### Recommendation 3–develop methods to recover viable agents and functional toxins from aerosols

Confirming the viability of an aerosolized pathogen is unnecessary for environmental monitoring in the *detect-to-warn* mode, but it is essential for surveillance in the *detect-to-treat* mode. Viability confirms that a BTA of interest was detected that can potentially cause infection. It also enables antibiotic susceptibility testing to confirm the appropriate antibiotic(s) for prophylaxis. An integrated system does not currently exist for routine surveillance of air borne organisms or toxins of interest *and* simultaneous confirmation of agent viability or toxicity. In currently existing environmental monitoring systems, for example, a 24-h period of collection results in cell desiccation and possibly additional stresses such as denaturation of proteins that pose substantial challenges for the recovery of viable organisms and active toxins. Given that surveillance in *detect-to-treat* mode can reduce morbidity and mortality to a much greater degree than in *detect-to-warn* mode, developing an effective approach to reduce the loss of viable pathogens and toxins during aerosol sampling would be a valuable investment.

Any approach to recovering and documenting viable, non-sporulating biological agents and active toxins of interest must address two major problems: (a) desiccation-related loss of viability or stability; and, (b) ability to rapidly distinguish a viable BTA of interest from other viable cells that may be present in a sample.

Although desiccation resulting in cellular destruction or protein denaturation is commonly believed to be the cause of cell death and loss of toxin activity in aerosol samples, dehydration is a common method used for long-term preservation of microbial cells or toxins. In aerosol samples, cell death may occur from the rapid re-hydration of cells during assays for viability (Crowe et al., 1992). During dehydration, the cell membrane transitions to a crystalline state. The disorderly transition of the membrane from a crystalline state back to a semi-fluid state during abrupt re-hydration can cause membrane leakage, resulting in cell death (Crowe et al., 1992). Thus, gentle re-hydration procedures may substantially increase recovery of viable cells in aerosol samples (Crowe et al., 1992). Use of membrane stabilizers or devices that promote gentle rehydration may substantially increase recovery of viable cells directly from aerosols or from aerosol collection filters. However, this requires further evaluation. Aerosol samplers that collect samples directly into a liquid medium may be an alternative solution if proper osmolarity can be maintained to prevent osmolysis. Rapidly distinguishing a viable BTA in an aerosol sample from other viable background microorganisms is a more challenging problem for which there is no obvious solution at present. Although the viability of vegetative cells can be determined rapidly by staining and microscopy, the BTAs in a complex mixture cannot be identified by cellular morphology. More broadly, the viability of BTAs can be determined by cultivation, but the lack of selective media that enable exclusive cultivation of BTAs means a screening method must be applied subsequently to detect viable BTAs in a background of other viable organisms.

### Recommendation 4–abandon outdoor monitoring and focus on indoor and special event monitoring

For an outdoor, city-wide environmental surveillance system to achieve an acceptable level of detection sensitivity, a denser spatial network of detectors is needed. A denser network would increase the likelihood of detection by: (i) increasing the probability that one or more detectors are close to the point source where the released agent concentrations are highest, and (ii) increasing the probability that at least one detector will capture an event despite changing air-flow patterns. A denser network would also improve the likelihood that multiple detectors document an event, which can facilitate the characterization of the magnitude and spatial coverage of an event. However, the cost of denser networks is expected to make outdoor environmental surveillance prohibitive. Consequently, the panel recommended abandoning outdoor monitoring because, as currently practiced, outdoor environmental monitoring seems unlikely to achieve its operational goal (Person and Currie, 2015) and therefore does not justify the cost.

Environmental monitoring indoors and at special events is more tractable owing to the more constrained spatial scale and is consistent with the scale of past attacks (Guillemin, 2011; National Research Council, 2011) and threat assessments. Current technology is more likely to succeed in these settings owing to the tighter spatial focus and the absence of wind, precipitation, and other environmental variability that can easily confound outdoor environmental monitoring. If an indoor or special event attack occurs, the greater proximity of detectors to the point source and a more controlled environment increases the probability of capturing a detectable amount of target material. It should be noted that shifting to indoor monitoring does not preclude the detection of large-scale outdoor releases because the composition of outdoor aerosols contributes to the composition of indoor aerosols (Meadow et al., 2014; Adams et al., 2015; Miletto and Lindow, 2015; Ruiz-Calderon et al., 2016; Wilkins et al., 2016).

### Recommendation 5–reduce monitoring costs by developing a reagent-less sensor that can triage aerosol samples in real-time, identifying a smaller set of ‘suspect aerosol samples that merit testing with conventional real-time PCR assays

The current concept of a 24-h collection period followed by laboratory-based analysis of samples by PCR creates unacceptable delays and costs. The delay in detection can be as long as 36 h, due to the aerosol collection period, retrieval, transport interval, and analysis time (Person and Currie, 2015). Automated sample collection and testing can reduce the time-to-results to 6 h, but is projected to substantially increase costs because of increased use of consumables for DNA extraction and PCR assays (Person and Currie, 2015), wear and tear of the platform from extensive use, and temperature regulation to maintain reagent integrity. To solve this problem, detectors are needed that reduce costs required for reagents, maintenance and replacements of units, and the extensive engineering to avoid reagent-damaging temperature variation. Reducing assay volumes through use of microfluidic is a potential incremental solution, although tradeoffs in detection sensitivity and potential clogging during movement of fluids would require evaluation. A transformative solution to reducing operational costs is the development of a system that includes a reagent-less, real-time detector able to triage aerosol samples to substantially reduce the number of samples that require sophisticated testing. This is a formidable technical challenge that requires continued investment.

Several approaches employing mass spectrometry (MS) have been suggested as potential technologies for a reagent-less bioagent sensor (National Research Council, 2005; National Academy of Sciences, 2011, 2013), but thus far no single method has proved effective in providing the specificity and accuracy needed for routine use. Furthermore, the data observed in many peer-reviewed efforts use simple mixtures or even pure samples of target organisms, which do not reflect the real-life situations where samples contain hundreds of organisms and diverse biological materials. The MS techniques that have been investigated include pyrolysis MS (Tripathi et al., 2001; Wilkes et al., 2006), pyrolysis-derivatization MS/MS (Griest et al., 2001), derivatization gas chromatography GC-MS (Jeong et al., 2014), aerosol laser ablation (LA) MS (Fergenson et al., 2004), and aerosol matrix assisted laser desorption/ionization (MALDI) MS (Jung et al., 2014). The high cost, generally large footprint, and power requirements (mostly because of vacuum pumps) are factors that have limited the application of MS in autonomous detection devices. At present, the MS-based methods have inadequate specificity for definitive identification as well as low sensitivity, which limits their use for environmental monitoring. The complexity of the microbial background in aerosols reduces detection specificity, and the potentially low numbers of target microorganisms affects the detection sensitivity in some MS formats. To date, the application of MS in environmental monitoring has generally focused on its use as a stand-alone, definitive identification tool. The group felt that this family of technologies has not been explored in a systematic way for use as a trigger—that is, a reagent-less device capable of sample triage, triggering the use of more sophisticated PCR analysis for a reduced set of suspect aerosol samples. If a cost effective and reagent-less MS instrument can be identified as a suitable trigger system, this option may warrant further investigation. A comprehensive study to summarize peer-reviewed literature for all known spectroscopic instrumentation for the detection of biological agents, including technical specifications for each, information about how well they work, and how economical they are when used in actual practice is in order. These data can then be used to assess how spectroscopic-based instruments could be integrated with current biological detection technologies, outlining the strengths and weaknesses of the detection mechanisms in the context of agent-agnostic detection and potential use of multiplexed (multiple types of spectroscopy combined) detection configurations for the detection of biological agents.

Raman spectroscopy has been evaluated as a potential technology platform for a reagent-less environmental monitoring system (Ronningen and Bartko, 2009; National Academy of Sciences, 2013; Kusić et al., 2014; Schultz et al., 2014; Zhou et al., 2014; Baritaux et al., 2015; Hlaing et al., 2016; Stöckel et al., 2016; Gao et al., 2017; Lorenz et al., 2017). This approach relies on the notion that target biological agents exhibit reproducible Raman profiles that are sufficiently distinct from the profiles of material routinely observed in aerosols, enabling triage of aerosol samples (i.e., discrimination of “suspect” aerosol samples from a large volume of non-relevant samples). In one example of a Raman-based aerosol-monitoring device, aerosol particles are collected through impaction onto a metallic tape and screened for Raman profiles of potential interest (Ronningen and Bartko, 2009; National Academy of Sciences, 2013). Fields with profiles of interest are further interrogated by Raman spectroscopy and compared against a proprietary reference database of biological threat agents, offering the possibility of rapid screening and extensive hands-free operation at low cost (Ronningen and Bartko, 2009; National Academy of Sciences, 2013). The principle of acquiring samples on continuous metal tapes is interesting because it facilitates the display of possible targets in a much less crowded field, reducing the number of interfering particles (other organisms) for spectral analysis. Additionally, since the impacted metallic tape is not destroyed, additional testing can be done using laboratory based methods (e.g., PCR and sequencing) and evidence preserved for future use. Like MS-based detectors, conventional Raman-based detectors require a large quantity of aerosolized target material for detection, which limits their value. While the speed and simplicity of Raman-based sample screening is very attractive, Raman or other spectroscopic platforms require extensive testing and evaluation to understand suitability for environmental monitoring in terms of its sensitivity, specificity and robustness. Such characterization should consist of carefully designed validation studies conducted with relevant biological materials under realistic field conditions, and include evaluation of both the primary (Raman) and confirmatory/secondary (PCR) assays. An unexplored concept is to augment a Raman platform with additional types of spectroscopy instrumentation to fill in the spectral blanks that occur from use of Raman spectroscopy alone.

## Conclusions

Our recommendations are consistent with those from prior expert panels that evaluated environmental surveillance for biodefense (National Research Council, 2005; National Academy of Sciences, 2011, 2013). Although a perfect surveillance system (immediate detection and event characterization, exquisite sensitivity, zero false positives or negatives) to detect biological agents of interest is not feasible owing to technical and resource limitations, a system to detect and mitigate the most serious attack scenarios is conceivable. A robust surveillance system requires a combination of public health surveillance and environmental monitoring. Public health surveillance can detect the full range of attack scenarios, but is generally viewed as insufficient to substantially mitigate consequences of bioweapons that rapidly induce illness and death. With suitable research to support the operational and technical recommendations above, a reliable environmental monitoring system can be developed to detect the most serious scenario, i.e., a large-scale bioaerosol release with a high fatality rate. The scenarios of greatest concern are those that target locations with high population density. High population densities are linked to specific facilities and special events. Therefore, a shift from general outdoor surveillance to specific indoor and special event surveillance is compelling and likely to increase successful detection. To perform environmental monitoring at an acceptable cost, it is essential to develop a triggered sensor that can greatly reduce the number of samples subjected to time-consuming and expensive assays. Proper validation of the system and transparency about detection efficacy can engender greater confidence of public health officials and other stakeholders.

### List of participants

Kevin Anderson, Department of Homeland Security; Tamilselvam Batcha, University of Illinois; Thomas Blake, Centers for Disease Control and Prevention; Steven Blanke, University of Illinois; Catherine Branda, Sandia National Laboratory; Katrina Campbell, Queen's University Belfast; Stephen Casalnuovo, Sandia National Laboratory; Rodrigo Castillo-Garza, United Technologies Research Center; Douglas Cerasoli, U.S. Army Medical Research Institute of Chemical Defense; Jean Challacombe, Los Alamos National Laboratory; James Dillman, US Army Medical Research Institute of Chemical Defense; John Dunbar, Los Alamos National Laboratory; David Franz, NGI; Mark Geisberg, Silver Lake Research Corporation; David Hodge, Department of Homeland Security; Robin Holland, University of Illinois at Urbana Champaign; Hayden Huang, Government Accountability Office; Molly Hughes, University of Virginia; Janine Hutchison, Pacific Northwest National Laboratory; Morten Jensen, Gate Scientific; Justin Kita, Federal Bureau of Investigation; Luther Lindler, Department of Homeland Security; Ronald Manginell, Sandia National Labs; Patrick Martin, Takara Bio USA; Patrick McNutt, U.S. Army Medical Research Institute of Chemical Defense; Eric Merkley, Pacific Northwest National Laboratory; Stephen Morse, Centers for Disease Control and Prevention; William Nelson, Tetracore; Tom O'Brien, Tetracore; Timothy Persons, Government Accountability Office; Segaran Pillai, HHS Food and Drug Administration; Kristian Scaboo, Gate Scientific; Shashi Sharma, HHS Food and Drug Administration; Sushil Sharma, Government Accountability Office; Patrick Stayton, University of Washington; Nicole Steinmetz, Case Western Reserve University; Jane Tang, Department of Homeland Security; Bernhard Weigl, Global Good; David Wunschel, Pacific Northwest National Laboratory.

These participants were attending a conference on Chemical and Bioterrorism Defense and were invited as subject matter experts to the independently organized discussions from which this perspectives article arose.

## Author contributions

All authors listed have made a substantial, direct and intellectual contribution to the work, and approved it for publication.

### Conflict of interest statement

The authors declare that the research was conducted in the absence of any commercial or financial relationships that could be construed as a potential conflict of interest.

## Footnotes

^1^Available online at: http://lunainc.com/forensic-swabs-perform-needed-criminal-investigations/
